# The Link Between Diabetes Mellitus and Tau Hyperphosphorylation: Implications for Risk of Alzheimer's Disease

**DOI:** 10.7759/cureus.18362

**Published:** 2021-09-28

**Authors:** Amy L Hobday, Mayur S Parmar

**Affiliations:** 1 Foundational Sciences, Nova Southeastern University Dr. Kiran C. Patel College of Osteopathic Medicine, Clearwater, USA

**Keywords:** alzheimer's disease, endocrinology and diabetes, amyloid beta, hyperglycemia, tauopathy

## Abstract

Diabetes mellitus (DM) is characterized by hyperglycemia caused by a lack of insulin, insulin resistance, or both. It is associated with the development of secondary complications resulting in several comorbidities. Recent studies have revealed an increased risk of developing cognitive dysfunction or dementia in diabetes patients. Diabetes mellitus is considered a risk factor for many neurodegenerative diseases, including Alzheimer's disease (AD). There is increasing evidence to support a link between DM and AD. Studies have shown the dysfunction of insulin signaling in the brain, resulting in increased tau protein phosphorylation (hyperphosphorylation), a hallmark and biomarker of AD pathology, leading to accumulation of neurofibrillary tangles. In DM, the insulin dysfunction in the brain is reported to alter the glycogen synthase kinase-3β (GSK-3β) activity showing to enhance tau phosphorylation. In DM and AD, GSK-3β signaling has been involved in the physiological and pathological processes, respectively. This potentially explains why DM patients have an increased risk of developing AD with disease progression and aging.

Interestingly, several in vivo studies with oral antidiabetic drugs and insulin treatment in DM have improved cognitive function and decreased tau hyperphosphorylation. This article will review the relationship between DM and AD as it relates to tau pathology. More understanding of the link between DM and AD could change the approach researchers and clinicians take toward both diseases, potentially leading to new treatments and preventative strategies in the future.

## Introduction and background

Diabetes mellitus (DM) is the most common endocrine and metabolic disease worldwide. As per the National Diabetes Statistics Report 2020, DM affects over 34.2 million Americans, with 1.5 million new cases reported in 2018. There are two common types of DM, type 1 DM (T1DM) and type 2 DM (T2DM). In T1DM, there is progressive destruction of pancreatic insulin-producing islet β-cells caused by the autoimmune assault, which results in insulin deficiency. Both genetic and environmental factors play important roles in the progression of T1DM. T2DM is characterized by dysfunction of islet β-cells, resulting in impaired insulin secretion and resistance to the actions of insulin in peripheral tissues (i.e., insulin resistance). Most diabetes mellitus individuals have at least one comorbid chronic disease and have higher risks of cardiovascular complications, end-stage renal disease, and hypertension [1,2]. The most common comorbid condition observed in T2DM patients includes hypertension, obesity, and hyperlipidemia. The comorbidity burden tended to increase in older age groups [2].

Diabetes mellitus is considered an independent risk factor for cognitive dysfunction or dementia, especially those closely related to Alzheimer's disease (AD) [3-5]. Alzheimer's disease is pathologically characterized by senile plaques formed of amyloid-β deposition outside the neuron and neurofibrillary tangles (NFTs) inside neurons, composed of hyperphosphorylated tau [4,6]. With the AD progression, these pathological neuronal changes result in neuronal destruction and cognitive impairment [4,6]. Clinically, there is a progressive loss of memory and a decline in cognitive functions in AD [6]. These include difficulties with memory, language, problem-solving, and other thinking skills that affect a person's ability to perform everyday activities. The dementia phase of AD typically progresses slowly in three general stages: mild, moderate, and severe [6]. An estimated 6.2 million Americans aged 65 and older are living with AD in 2021 [6].

Due to the increased risk of late-onset AD in diabetes mellitus, some scholars have referred to AD as type 3 diabetes mellitus [7,8]. Interestingly, the presence of DM nearly doubles an individual's risk of developing AD [9]. Increasing evidence has shown that insulin plays a role in AD, and brain glucose metabolism has been impaired in AD patients. Several studies have highlighted the dysfunction of insulin signaling-related mechanisms underlying the increased risk of AD in DM [4,10,11]. Tau hyperphosphorylation, a major component of paired helical filaments in NFTs found in the AD brain, has been associated with deficient brain insulin signaling in DM [4,12]. Glycogen synthase kinase-3β (GSK-3β) is the common kinase in insulin signaling transduction and tau hyperphosphorylation. Hence, it is believed that dysfunction of insulin signaling in the DM that modulates the GSK-3β pathway may lead to increased tau hyperphosphorylation, resulting in an increased risk of developing AD. Ongoing studies highlighting the insulin/GSK-3β dysfunction and tau hyperphosphorylation could potentially explain the relation and potential links between DM and AD. However, along with the above underlying cause, several mechanisms are not well understood.

In this article, we reviewed the relationship between DM and AD as it relates to the tau pathology, a hallmark and biomarker of AD pathology. We also focus on understanding the role of insulin and GSK-3β signaling in the brain in relation to tau hyperphosphorylation. The reported studies will include findings from both in vivo animal and human studies. We further reviewed the pharmacological treatments targeting DM and its effect on tau pathology underlying AD.

## Review

Insulin signaling in the brain and role in tau hyperphosphorylation

The role of insulin in the brain is different than that of insulin in the periphery. In the brain, insulin has limited glucose metabolism effects and greater effects on neuronal function, including synaptic plasticity, learning, and memory [13]. Insulin is vital for many neurological pathways in the brain, including insulin's ability to promote neuronal regeneration [14]. Insulin is also a critical factor in releasing many neurotransmitters, including those essential for memory formation, such as acetylcholine, norepinephrine, and epinephrine [3]. It stimulates amino acids reuptake, while also a key stimulator of membrane ion transport and hyperpolarization [14]. Studies in animal models and humans have reported a mechanistic interplay between disrupted insulin signaling and AD pathogenesis [15-17]. These could be related to impaired insulin signaling in the brain resulting in tau hyperphosphorylation, one of the pathological features in AD. Several studies have shown insulin to regulate tau phosphorylation [11,18,19].

Tau is a microtubule-associated cytosolic protein expressed in nerve cells. Under normal physiological conditions, tau promotes microtubule assembly and stabilization, which is important for anterograde axonal transport. In certain pathological conditions, such as AD and primary tauopathies, tau proteins undergo modifications, mainly through phosphorylation resulting in the aberrant aggregates of hyperphosphorylated tau proteins. Under physiological and pathological conditions, tau phosphorylation is regulated by several kinases, such as GSK-3β, cyclin-dependent kinase 5 (CDK5), mitogen-activated protein kinase/extracellular-signal-regulated kinase (MAPK/ERK), and c-Jun N-terminal kinase (JNK) [11,12]. The protein phosphatase 2A (PP2A) is considered to be the major tau phosphatase essential for regulating tau phosphorylation [11]. The hyperphosphorylation of tau prevents tau binding to microtubules. Accumulation of intraneuronal neurofibrillary tangles (NFTs), composed of hyperphosphorylated tau protein assembled in paired helical filaments (PHFs) in the brain, is strongly correlated with neurodegeneration and clinical signs of dementia [11].

A significant negative correlation between tau phosphorylation and insulin activation and the levels of insulin signaling pathway components has been observed [19]. In the brain, when insulin binds to its receptor, there is rapid autophosphorylation of the tyrosine kinase insulin receptor, leading to its activation. When activated, the insulin receptor recruits and phosphorylates many different insulin receptor substrates (IRS-1), including a tyrosine-phosphorylated IRS-1, which displays a binding site for phosphatidylinositol 3-kinase (PI3K). The activation of PI3K, in turn, activates mitogen-activated protein kinase (MAPK) and protein kinase B (AKT) through Thr308 phosphorylation, which targets GSK-3β, a prominent tau kinase. The phosphorylation of GSK-3β by AKT signaling results in its inactivation leading to no GSK-3β mediated tau phosphorylation [19]. This pathway is halted when insulin signaling is impaired or absent, leading to no phosphorylation of GSK-3β kinase. This impaired insulin signaling thereby results in GSK-3β-mediated tau hyperphosphorylation.

Conversely, tau aggregation has also been reported to impair insulin signaling in the brain [20]. This could mean that in the disease state with impaired insulin signaling in the brain, where tau hyperphosphorylation occurs, the aggregated tau could further contribute to insulin signaling dysfunction, resulting in cognitive decline. However, the influence of tau pathology on insulin signaling is not completely well understood and needs further evaluation.

Insulin dysfunction can also disrupt MAPK and AKT signaling pathways known to regulate tau protein functioning [21]. Tau protein is also inversely regulated through modification by O-GlcNAcylation. This is downregulated during decreased brain glucose metabolism directly related to functional insulin presence within the brain. This down-regulation leads to hyperphosphorylation of the tau protein [19]. Overall, impairment of insulin signaling is directly associated with tau phosphorylation. Interestingly, in postmortem AD and other tauopathies, insulin is accumulated as oligomers in the hyperphosphorylated tau-containing neurons [22]. A significant increase of tau phosphorylation at the AT180 epitope was observed in an insulin knockout animal model [11].

PP2A and tau

Inhibition of protein phosphatase 2A (PP2A) in human AD patients seems to be an important factor in the disease progression, as PP2A is considered the major tau phosphatase essential for regulating tau phosphorylation [11]. In AD, the deregulation of the PP2A protein can override the over-activation of any kinases, as the PP2A protein dephosphorylates the tau protein at all its epitomes [11]. It has been shown that alteration in glucose metabolism results in abnormal tau hyperphosphorylation due to inhibition of PP2A activity in the brain induced by hypothermia [11,12]. Hypothermia is a common outcome observed in several human diabetic populations [11]. Hence, another potential underlying mechanism for the increased tau hyperphosphorylation, thereby increased risk of dementia and AD, in the diabetic condition could be related to PP2A inhibition.

Alzheimer's disease and diabetes mellitus link: human studies

The risk of developing dementia, especially AD, is 1.4-4.3 times higher in diabetic versus non-diabetic individuals [13]. Several epidemiological studies have shown an association between T2DM and the increased risk of AD. The suggested mechanisms for this association include insulin resistance, insulin deficiency, impaired insulin receptor, glucose toxicity, vascular inflammation, and cerebrovascular injury [23]. Other mechanisms include an imbalance between production and clearance of amyloid-β, abnormal tau phosphorylation, and structural and functional neuron damage, leading to a lack of neurotransmitters and IL-6 abnormalities [5].

Postmortem studies have shown an increase in hyperphosphorylated tau in patients with DM compared to age-matched controls suggesting a link between DM and AD [18]. Additionally, western blot analysis has shown a significant increase in the total tau protein present in human patients with both DM and AD, in addition to non-significant hyperphosphorylation at the Ser202/Thr205 and pThr231 epitopes [18]. It has also been shown that insulin resistance increases as we age, leading to more susceptibility to diseases like AD [12]. Due to the increased insulin resistance in DM, it may amplify AD progression and lead to pathogenesis earlier and more rapidly. Moreover, neuronally derived exosomes of total plasma from AD subjects show an increase in pIRS-1Ser312, a marker for insulin resistance 10 years before the clinical onset of AD [20]. This observation indicates a predisposition to insulin resistance in those with AD, which correlates with AD pathology.

The presence of the apolipoprotein E (*APOE*)* e4* allele markedly increases the risk of developing AD and an earlier age of onset for developing the disease [24]. The presence of the *APOE e4 *allele has been of great interest as it seems to predict a higher susceptibility to developing AD in one's life. It has been shown that an increase in systemic insulin resistance, characteristic of DM, is associated with higher cerebrospinal fluid phosphorylated and total tau levels in asymptomatic *APOE e4 *carriers [25]. Further, those with the *APOE e4* allele suffering from DM and AD have shown a stronger correlation in a decrease in glucose metabolism than controls [3]. This process has been linked to DM and AD separately, but the association with the *APOE e4* allele and the comorbidity of DM and AD presents a stronger correlation [3].

A significant negative correlation between insulin signaling pathway and calpain 1 activation has been reported [19]. Calpain 1 is a calcium-activated intracellular proteinase, which degrades many proteins within the brain, including tyrosine kinases like the insulin receptor [19,26]. As we age, the brain is subject to increased oxidative stress and increased activation of N-methyl-D-aspartate (NMDA), which leads to an increase in calcium concentration in the brain. In turn, the rise in calcium levels increases the activity of the calpain enzymes, leading to an increase in the degradation of many proteins, including tyrosine kinases [19,26]. Calpain has also been shown to activate CDK5 and GSK-3β kinases, which are involved in tau hyperphosphorylation [26]. Therefore, an individual with DM who is suffering from insulin impairment will not only have a downregulation of tyrosine kinases due to negative feedback but a greater risk of tau hyperphosphorylation due to decreased insulin significantly impairing GSK-3β signaling. Before these individuals age, they already suffer from the damage caused by the calpain proteinases that come with natural aging. Hence, as they age, their risk for adverse effects and pathology associated with these proteins increases. This finding could explain why the average age of dementia diagnosis in those with T1DM is 64.6 years old. In comparison, the average age of diagnosis of dementia in the overall population is 83.7 years old [27,28].

Finally, compared with dementia in the healthy population, dementia in the diabetic population shows significantly more tau accumulation than amyloid-β. In fact, upon positron emission tomography (PET) imaging, 91% of patients with diabetes-related dementia showed an increase in tau protein, while only 40% showed an accumulation of amyloid-β [29]. This finding indicates that tau pathology could be the link between diabetic patients and developing AD.

Alzheimer's disease and diabetes mellitus link: in vivo rodent studies

In mice, the link between AD and DM has been more extensively studied than in humans. By injecting streptozotocin, a chemical that causes insulin deficiency by destroying the insulin-producing beta cells in the pancreatic islets of Langerhans, into mice to induce hyperglycemia, many studies have shown correlations between DM and AD. Mice injected with streptozotocin show a significant increase in tau hyperphosphorylation at the S422 epitope, one of AD's major pathological sites [9]. Also, insulin and its growth factors have been shown to play a role in neuron protection against the toxicity of the amyloid-β and tau pathologies [30]. The loss of insulin in DM then leaves the neurons vulnerable to toxicity by these pathological features. Using streptozotocin, Santos et al. considered mice hyperglycemic if they had a blood glucose level above 250 mg/dL three days post-drug administration. Significant bodyweight reduction and significant elevation in hemoglobin A1c (HbA1c) concentration compared to controls were also used to solidify the T1DM model. It was found that those mice which were hyperglycemic had exacerbated tau protein hyperphosphorylation, especially at Ser396 epitope, one of the earliest sites known in AD. The hyperphosphorylation at the Ser396 epitope is associated with more fibrillogenic tau and is primarily responsible for the functional loss of the tau-mediated tubulin polymerization [30]. By identifying hyperglycemia through a fasting plasma glucose, greater than or equal to 16.7 mmol/L 72 hours post-drug administration, tau protein levels were significantly greater in the cerebrospinal fluid (CSF) of those considered hyperglycemic than controls on days 60 and 75. There was also a significant negative correlation with tau protein phosphorylation and acetylcholine levels, indicating impaired NMDA receptor activity. This was further strengthened by a substantial increase in reference memory errors for the hyperglycemic mice than healthy controls [5].

In another T2DM rat model of hyperglycemia, it was shown that the rats in the T2DM + AD group (composite animal model) showed significant changes in learning and memory when compared to control and those with just T2DM or AD [31]. In this study, a single intraperitoneal injection of streptozotocin (30 mg/kg) along with the provision of a high-fat and high carbohydrate diet for eight weeks was used to generate the T2DM rat model [31]. The composite animal model (T2DM + AD) was established by injecting Aβ1-40 into the bilateral hippocampus at stereotaxic coordinates in the T2DM rat model [31]. There was a significant difference between hyperphosphorylated tau in the AD and T2DM + AD groups compared to the control and T2DM groups alone. In the hippocampal tissues of the T2DM + AD group, a significant increase in the hyperphosphorylated and total tau in the hippocampal tissue was observed compared to all other groups. The hippocampal hyperphosphorylation was not significantly different between the control and T2DM groups [31]. In the same study, a significant difference in the mammalian target of rapamycin (mTOR) was observed between control and AD groups and T2DM and T2DM + AD groups. mTOR, an essential protein involved in insulin resistance, is a signaling protein that triggers insulin sensitivity adjustments through feedback. The protein is also responsible for maintaining beta-cell function in the islets of Langerhans. Overactivation of mTOR has also been linked to increased neurotoxic hyperphosphorylation [31]. A significant difference in mTOR signaling was also observed between the T2DM and T2DM + AD groups. These two groups also showed a significant increase in the mTOR in the hippocampal tissues compared to control and AD groups; further, the T2DM + AD group has significantly more than the DM group [31]. This difference indicates an increased presence of reduced insulin regulation in those patients with DM and AD, as the progression to both diseases correlates with a prominent increase in mTOR signaling, which is crucial in adjusting insulin sensitivity.

In a study involving the nonobese diabetic (NOD) mice, which spontaneously develop type 1 diabetes at 13-25 weeks due to a mutation that destroys insulin-producing beta cells, tau hyperphosphorylation was significantly increased compared to controls at the S422 epitope [32]. In the NOD mice, inhibition of protein phosphatase 2A (PP2A) activity was also noted, which correlated with the elevated tau phosphorylation [32]. Deregulation of PP2A in the NOD mice is the likely possible mechanism contributing to the AD-like tau hyperphosphorylation in the brain [32]. This was seen in adult NOD mice but not in the young NOD mice [32]. Overall, this animal model study also highlighted diabetes being a risk factor for developing tau hyperphosphorylation, the underlying pathology in AD.

Antidiabetic drugs: potential benefit in reducing the risk of dementia and Alzheimer's disease

Due to the strong associations that have been drawn between AD and DM, it is imperative to examine the drugs used to treat these diseases. The focus is toward those drugs used to treat DM, as it is the primary condition these patients face and treated. For instance, rosiglitazone, a drug used to treat DM, has been shown to increase insulin sensitivity and, in turn, increase the memory and attention of those with AD but without the *APOE e4 *allele [33]. Further, in an animal model of diabetes, rosiglitazone treatment resulted in a reduction in tau phosphorylation [34]. Using an antidiabetic drug to improve AD symptoms shows a strong link between the two disease processes. Also, treatment with regular insulin has been shown to block the mild beginnings of tau hyperphosphorylation [12], indicating the importance of insulin in regulating tau and preventing AD progression. Insulin treatment has also been shown to partially reverse the Ser396 pTau/total tau hyperphosphorylation ratios [30]. Insulin sensitizer drugs have been shown to reduce the hyperphosphorylation of tau through inhibiting the action of GSK-3β, but this treatment does not increase the levels of insulin in the brain [13]. Due to this, these treatments may not reduce all adverse outcomes of impaired insulin function in the diseased brain as the normal insulin levels and signaling maybe not be restored.

Interestingly enough, experiments with intranasal insulin have begun to show an increase in CSF insulin without increasing peripheral insulin [11]. These findings may benefit those with DM whose treatment is working in the periphery but lacking in the brain. Also, this treatment can help combat all effects of decreased functional insulin in the CNS.

Metformin is currently the first-line drug treatment for T2DM and is the first prescribed drug for uncontrolled T2DM. In T2DM, metformin use decreased the risk of developing dementia by 24% compared to their counterparts who were not treated with this drug. In comparison, treatment with sulfonylureas only reduced the risk of dementia by 15% [35]. Interestingly, when these two drugs were combined, there was a reduced risk of developing dementia over eight years of 35% [35]. Further, when looking at a sample of 145,928 individuals over the age of 60, individuals with diabetes treated with pioglitazone, a thiazolidinedione, for the long-term showed a lower dementia incidence. In addition, those who were not treated with this drug had a 23% increased risk of developing dementia. Interestingly, in diabetes patients, short-term use of pioglitazone was associated with similar AD risk as that in non-diabetic patients [35].

These findings suggest the potential risk of dementia in DM patients and how the antidiabetic drugs can be beneficial in controlling hyperglycemia and benefiting the patient in reducing their risk of developing dementia. More clinical trials and observational studies need to be done to elucidate the potential beneficial effects of antidiabetic drugs in lowering AD pathology and dementia risk. As mentioned above, there is growing evidence of several antidiabetic drugs for their ability to reduce the risk of dementia [35]. Abnormal tau hyperphosphorylation has been consistently shown to correlate well with cognitive decline and severity of dementia in AD [36-38]. It will be important to know whether the antidiabetic drugs' ability to reduce the risk of dementia in DM and AD is related to the reduction in tau hyperphosphorylation.

Alzheimer's disease risk in type 1 versus type 2 diabetes mellitus

The comparison of T1DM to T2DM is imperative when discussing the progression to AD as the individual relationships seem to contradict each other. It has been shown that in hyperglycemic mice treated with streptozotocin (T1DM model), the hyperphosphorylation of tau leads to hippocampal learning and memory deficits, which correlates with the research [18]. However, the hyperglycemic mice of a T2DM model showed impairment in learning independently to the hyperphosphorylation of tau when compared to controls. Further, these mice showed learning deficits whether they presented with normal tau, hyperphosphorylated tau, or had a tau knockout. The T2DM mouse model also showed a significant increase in inflammation in the hippocampus, in alliance with increased interleukin 10 (IL-10), interleukin 6 (IL-6), and tumor necrosis factor (TNF)-alpha, but this process was independent of tau hyperphosphorylation [18]. These findings beg the question, is tau hyperphosphorylation only a risk factor for AD in those with T1DM? Is insulin loss a trigger for tau hyperphosphorylation, while insulin resistance does not contribute to the full pathology? With age, the insulin begins to decrease in the body due to an overworked pancreas failing to produce any more insulin [11]. This stage of T2DM strongly mirrors T1DM. Ameliorating the brain insulin resistance and dysfunction in a T2DM patient as disease progress will be crucial to prevent the risk of cognitive impairment, dementia, and AD.

Insulin crosses the blood-brain barrier (BBB) and enters the brain by a saturable receptor-mediated process [39,40]. Prolonged hyperinsulinemia, often associated with T2DM, leads to a down-regulation of insulin receptors in the BBB and reduction in insulin transport into the brain [11,41]. This could further contribute to central insulin resistance and poor insulin levels in the brain and CSF, mirroring T1D. It may be at this stage in T2DM that the hyperphosphorylation of tau begins to emerge, leading to an increased risk of AD and other cognitive deficits. Moreover, individuals with T1DM with a poorly controlled disease process, characterized as an HbA1c of 8 or greater, were shown to have an increased risk of developing dementia than those whose disease process was adequately controlled [27]. This indicates that insulin loss in both T1DM and T2DM plays a role in brain health, mainly memory functions. Due to this, treatments such as intranasal insulin may be pertinent in treating those with T2DM.

## Conclusions

The literature review suggests that in diabetes, dysfunction of insulin and altered GSK-3β signaling in the brain contribute to the tau hyperphosphorylation, thereby potentially increasing the risk of developing AD. In diabetes mellitus, several other contributing factors for induction of tau pathology may include inflammation and increased inflammatory mediator levels in the brain. In addition, it is crucial to look at the relationship between DM, AD, and the *APOE e4* allele. The literature review suggests that oral antidiabetic medications have the potential to reduce tau hyperphosphorylation. Moreover, the treatment of T2DM by increasing insulin in the CSF could potentially prevent AD development. These findings have contributed to the preclinical and clinical evaluation of antidiabetic therapies to reduce AD pathology and improve cognitive performance. Overall, an enhanced understanding of the link between DM and AD could change the approach researchers and clinicians take toward both diseases, potentially leading to new preventative strategies in the future.

## References

[1] Piette JD, Kerr EA (2006). The impact of comorbid chronic conditions on diabetes care. Diabetes Care.

[2] Nowakowska M, Zghebi SS, Ashcroft DM (2019). The comorbidity burden of type 2 diabetes mellitus: patterns, clusters and predictions from a large English primary care cohort. BMC Med.

[3] Sun Y, Ma C, Sun H (2020). Metabolism: a novel shared link between diabetes mellitus and Alzheimer's disease. J Diabetes Res.

[4] Zhang Y, Huang NQ, Yan F, Jin H, Zhou SY, Shi JS, Jin F (2018). Diabetes mellitus and Alzheimer's disease: GSK-3β as a potential link. Behav Brain Res.

[5] Zhou Y, Zhao Y, Xie H, Wang Y, Liu L, Yan X (2015). Alteration in amyloid β42, phosphorylated tau protein, interleukin 6, and acetylcholine during diabetes-accelerated memory dysfunction in diabetic rats: correlation of amyloid β42 with changes in glucose metabolism. Behav Brain Funct.

[6] The Alzheimer's Association (2021). 2021 Alzheimer's disease facts and figures. Alzheimers Dement.

[7] Cai Z, Yan Y, Wang Y (2013). Minocycline alleviates beta-amyloid protein and tau pathology via restraining neuroinflammation induced by diabetic metabolic disorder. Clin Interv Aging.

[8] de la Monte SM, Wands JR (2008). Alzheimer's disease is type 3 diabetes—evidence reviewed. J Diabetes Sci Technol.

[9] Ke YD, Delerue F, Gladbach A, Götz J, Ittner LM (2009). Experimental diabetes mellitus exacerbates tau pathology in a transgenic mouse model of Alzheimer's disease. PLoS One.

[10] Tumminia A, Vinciguerra F, Parisi M, Frittitta L (2018). Type 2 diabetes mellitus and Alzheimer's disease: role of insulin signalling and therapeutic implications. Int J Mol Sci.

[11] El Khoury NB, Gratuze M, Papon MA, Bretteville A, Planel E (2014). Insulin dysfunction and tau pathology. Front Cell Neurosci.

[12] Planel E, Tatebayashi Y, Miyasaka T (2007). Insulin dysfunction induces in vivo tau hyperphosphorylation through distinct mechanisms. J Neurosci.

[13] Hu SH, Jiang T, Yang SS, Yang Y (2013). Pioglitazone ameliorates intracerebral insulin resistance and tau-protein hyperphosphorylation in rats with type 2 diabetes. Exp Clin Endocrinol Diabetes.

[14] Baskin DG, Figlewicz DP, Woods SC, Porte D Jr, Dorsa DM (1987). Insulin in the brain. Annu Rev Physiol.

[15] Benedict C, Grillo CA (2018). Insulin resistance as a therapeutic target in the treatment of Alzheimer's disease: a state-of-the-art review. Front Neurosci.

[16] Moloney AM, Griffin RJ, Timmons S, O'Connor R, Ravid R, O'Neill C (2010). Defects in IGF-1 receptor, insulin receptor and IRS-1/2 in Alzheimer's disease indicate possible resistance to IGF-1 and insulin signalling. Neurobiol Aging.

[17] Craft S, Claxton A, Baker LD (2017). Effects of regular and long-acting insulin on cognition and Alzheimer's disease biomarkers: a pilot clinical trial. J Alzheimers Dis.

[18] Trujillo-Estrada L, Nguyen C, da Cunha C (2019). Tau underlies synaptic and cognitive deficits for type 1, but not type 2 diabetes mouse models. Aging Cell.

[19] Liu Y, Liu F, Grundke-Iqbal I, Iqbal K, Gong CX (2011). Deficient brain insulin signalling pathway in Alzheimer's disease and diabetes. J Pathol.

[20] Gonçalves RA, Wijesekara N, Fraser PE, De Felice FG (2019). The link between tau and insulin signaling: implications for Alzheimer's disease and other tauopathies. Front Cell Neurosci.

[21] Gabbouj S, Ryhänen S, Marttinen M (2019). Altered insulin signaling in Alzheimer's disease brain - special emphasis on PI3K-Akt pathway. Front Neurosci.

[22] Rodriguez-Rodriguez P, Sandebring-Matton A, Merino-Serrais P (2017). Tau hyperphosphorylation induces oligomeric insulin accumulation and insulin resistance in neurons. Brain.

[23] Luchsinger JA, Tang MX, Stern Y, Shea S, Mayeux R (2001). Diabetes mellitus and risk of Alzheimer's disease and dementia with stroke in a multiethnic cohort. Am J Epidemiol.

[24] Zhao L, Gottesdiener AJ, Parmar M (2016). Intracerebral adeno-associated virus gene delivery of apolipoprotein E2 markedly reduces brain amyloid pathology in Alzheimer's disease mouse models. Neurobiol Aging.

[25] Starks EJ, Patrick O'Grady J, Hoscheidt SM (2015). Insulin resistance is associated with higher cerebrospinal fluid tau levels in asymptomatic APOEɛ4 carriers. J Alzheimers Dis.

[26] Mahaman YA, Huang F, Kessete Afewerky H, Maibouge TM, Ghose B, Wang X (2019). Involvement of calpain in the neuropathogenesis of Alzheimer's disease. Med Res Rev.

[27] Lacy ME, Gilsanz P, Karter AJ, Quesenberry CP, Pletcher MJ, Whitmer RA (2018). Long-term glycemic control and dementia risk in type 1 diabetes. Diabetes Care.

[28] Fishman E (2017). Risk of developing dementia at older ages in the United States. Demography.

[29] Hanyu H, Shimizu S, Sakurai H, Ishii K (2017). Amyloid and tau pet in diabetes-related dementia. Innov Aging.

[30] Santos RX, Correia SC, Alves MG (2014). Insulin therapy modulates mitochondrial dynamics and biogenesis, autophagy and tau protein phosphorylation in the brain of type 1 diabetic rats. Biochim Biophys Acta.

[31] Ma YQ, Wu DK, Liu JK (2013). mTOR and tau phosphorylated proteins in the hippocampal tissue of rats with type 2 diabetes and Alzheimer's disease. Mol Med Rep.

[32] Papon MA, El Khoury NB, Marcouiller F (2013). Deregulation of protein phosphatase 2A and hyperphosphorylation of τ protein following onset of diabetes in NOD mice. Diabetes.

[33] Risner ME, Saunders AM, Altman JF (2006). Efficacy of rosiglitazone in a genetically defined population with mild-to-moderate Alzheimer's disease. Pharmacogenomics J.

[34] Yoon SY, Park JS, Choi JE, Choi JM, Lee WJ, Kim SW, Kim DH (2010). Rosiglitazone reduces tau phosphorylation via JNK inhibition in the hippocampus of rats with type 2 diabetes and tau transfected SH-SY5Y cells. Neurobiol Dis.

[35] Bendlin BB (2019). Antidiabetic therapies and Alzheimer disease. Dialogues Clin Neurosci.

[36] Haroutunian V, Davies P, Vianna C, Buxbaum JD, Purohit DP (2007). Tau protein abnormalities associated with the progression of Alzheimer disease type dementia. Neurobiol Aging.

[37] Giannakopoulos P, Herrmann FR, Bussière T (2003). Tangle and neuron numbers, but not amyloid load, predict cognitive status in Alzheimer's disease. Neurology.

[38] Wesseling H, Mair W, Kumar M (2020). Tau PTM profiles identify patient heterogeneity and stages of Alzheimer's disease. Cell.

[39] Begg DP (2015). Insulin transport into the brain and cerebrospinal fluid. Vitam Horm.

[40] Banks WA, Jaspan JB, Kastin AJ (1997). Selective, physiological transport of insulin across the blood-brain barrier: novel demonstration by species-specific radioimmunoassays. Peptides.

[41] Wallum BJ, Taborsky GJ Jr, Porte D Jr (1987). Cerebrospinal fluid insulin levels increase during intravenous insulin infusions in man. J Clin Endocrinol Metab.

